# Insights Into the Association Between Stroke and Sarcopenia Risk in Adults Aged ≥ 50 Years: Cross‐Sectional Evidence From Two Large Population Longitudinal Cohorts

**DOI:** 10.1002/brb3.70763

**Published:** 2025-08-12

**Authors:** Xin Wang, Jie Zhang, Jing Xu, Ting Li, Qing Ye

**Affiliations:** ^1^ Center For Rehabilitation Medicine, Rehabilitation & Sports Medicine Research Institute of Zhejiang Province, Department of Rehabilitation Medicine, Zhejiang Provincial People's Hospital (Affiliated People's Hospital) Hangzhou Medical College Hangzhou People's Republic of China

**Keywords:** CHARLS, cross‐sectional study, HRS, sarcopenia, stroke

## Abstract

**Background and aim:**

Evidence has already shown that sarcopenia is linked to an elevated risk of cardiovascular disease (CVD). However, the association between stroke and sarcopenia risk remains underexplored in stroke and non‐stroke populations. This study investigated the stroke–sarcopenia relationship.

**Methods:**

We conducted a cross‐sectional analysis using data from Wave 3 (2015–2016) of the China Health and Retirement Longitudinal Study (CHARLS) and Wave 14 (2018–2019) of the Health and Retirement Study (HRS), focusing on ≥ 50 years adults. Missing data were imputed using the Python miceforest package with a random forest algorithm. All types of stroke were identified via self‐reported records where individuals had received a confirmed diagnosis from a physician. Sarcopenia status was evaluated using muscle strength (evaluated with grip strength in CHARLS and HRS), muscle mass (evaluated with appendicular skeletal muscle), and physical performance (evaluated with gait speed, the five‐repetition chair test, and the short physical performance battery). Logistic regression models estimated odds ratios (ORs) and 95% confidence intervals (CIs) for the primary analysis, with generalized estimating equation (GEE) models employed in sensitivity analyses to ensure result robustness.

**Results:**

The study comprised 16,859 participants from CHARLS (51% female, mean age 62.9 years) and 16,692 from HRS (41% female, mean age 67.6 years). In CHARLS, stroke was associated with significantly increased risks of possible sarcopenia (OR: 2.07, 95% CI: 1.49–2.99), sarcopenia (OR: 1.93, 95% CI: 1.23–3.06), and severe sarcopenia (OR: 3.42, 95% CI: 2.22–5.34). In HRS, stroke was linked to elevated risks of possible sarcopenia (OR: 3.11, 95% CI: 2.75–3.52) and severe sarcopenia (OR: 2.20, 95% CI: 1.24–3.65). Self‐care ability, nutritional status, and inflammatory factors were identified as key mediators, with findings remaining consistent across subgroups and sensitivity analyses.

**Conclusions:**

Stroke was associated with increased risk for sarcopenia and its severe forms in adults aged ≥ 50 years. We recommend sarcopenia risk evaluation in poststroke patients with accessible tools, also emphasizing that functional rehabilitation, nutritional optimization, and inflammation control hold promise to alleviate sarcopenia risk and enhance long‐term outcomes.

## Graphical abstract

The graph abstract text goes: In CHARLS and HRS, we showed RCS curves to investigate key factors in stroke‐sarcopenia association. CRP, nutritional status and physical function could be potential mediators. These findings confer some understandings on the mechanisms in stroke‐sarcopenia association, also offer opportunities for proper intervention to prevent post‐stroke sarcopenia. Abbreviations: RCS, restricted cubic spline; CRP, C‐reactive protein; IADL, instrumental activities of daily living; BMI, body mass index; OR, odd ratio; CI, confidence interval. SA, sarcopenia, PSA, possible sarcopenia, SSA, serve sarcopenia.

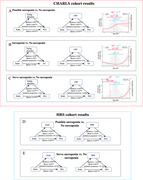

**Abbreviations**
AI: artificial intelligence
ASM: appendicular skeletal muscle
AWGS: Asian Working Group for Sarcopenia
BMI: body mass index
CHARLS: China Health and Retirement Longitudinal Study
CI: confidence interval
CRP: C‐reactive protein
CVD: cardiovascular disease
DXA: dual‐energy x‐ray absorptiometry
EWGSOP: European Working Group on Sarcopenia in Older People
GEE: generalized estimating equation
GLM: generalized linear model
HRS: Health and Retirement Study
IADL: instrumental activities of daily living
IDF: International Diabetes Federation
IQR: interquartile range
MET: metabolic equivalent of task
MetS: metabolic syndrome
MICE: multiple imputation by chained equations
NCEP/ATP III: National Cholesterol Education Program Adult Treatment Panel III
OR: odds ratio
RCS: restricted cubic spline
SD: standard deviation
SPPB: short physical performance battery
STROBE: Strengthening the Reporting of Observational Studies in Epidemiology
TyG: triglyceride‐glucose
VIF: variance inflation factor

## 1 Introduction

Sarcopenia is an age‐related condition marked by the progressive decline of skeletal muscle mass and function, contributing to increased risks of falls, fractures, and loss of independence, alongside substantial socioeconomic burdens (Sayer and Cruz‐Jentoft 2022; Cruz‐Jentoft 2019; Papadopoulou 2020). Over the past decade, consensus panels have refined diagnostic criteria for sarcopenia. The European Working Group on Sarcopenia in Older People (EWGSOP) established initial guidelines in 2010, updated in 2018 as EWGSOP2 for European populations (Cruz‐Jentoft et al. 2010, 2019), while the Asian Working Group for Sarcopenia (AWGS) released its 2019 consensus for Asian populations (L.‐K. Chen et al. 2014, 2020). These frameworks have standardized diagnostic thresholds, enhancing awareness, care, and research across diverse demographics.

Aging is a primary driver of cardiovascular disease (CVD), a leading cause of mortality among older adults (Arnett et al. 2019a, 2019b). Globally, CVD deaths rose by 14.5% from 2006 to 2016, though age‐standardized mortality declined by the same percentage (GBD 2016 Causes of Death Collaborators 2017; Dunbar et al. 2018). Ischemic stroke, a significant CVD subtype, possesses a substantial health burden, driving mortality and disability worldwide (Hilkens et al. 2024; Ruff et al. 2024). In China, stroke prevalence among adults aged ≥ 40 years was estimated at 2.6% in a cohort of 676,394 individuals. Despite reduced mortality due to effective prevention and early intervention, many stroke survivors face chronic disability, diminishing quality of life, and escalating healthcare costs (Tu et al. 2023; Wang et al. 2017; GBD 2019 Stroke Collaborators 2021). Large‐scale epidemiological data are essential to optimize stroke management and improve patient outcomes.

Poststroke patients frequently exhibit reduced muscle mass and strength, attributed to poor oral health, biological denervation, and chronic inflammation (Su et al. 2020; Shiraishi et al. 2018; Scherbakov et al. 2015). Stroke and sarcopenia often coexist, forming a vicious cycle that exacerbates both conditions (Springer et al. 2014). Experimental evidence suggested that stroke triggers catabolic pathways, reducing muscle mass within 3 weeks, with up to 24% loss in hemiplegic limbs within 6–12 months (Ryan et al. 2011). However, these findings are limited to small sample size and single‐center studies with uncertain adherence to standardized diagnostic criteria, and their applicability to older adults remains unclear. Given prior evidence linking sarcopenia to heightened CVD risk, including stroke (Gao et al. 2022; Qiu et al. 2024; J. Zhang, Jia, et al. 2023; X. Zhang, Ding, et al. 2023), we hypothesized that stroke is linked to elevated sarcopenia risk. To address limitations in previous research, we conducted a cross‐sectional analysis using two large longitudinal cohorts to examine the stroke–sarcopenia relationship in adults aged ≥ 50 years, estimating nutritional and inflammatory mediators and performing detailed subgroup analyses.

## 2 Methods and Materials

### 2.1 Study Population

Data were sourced from the China Health and Retirement Longitudinal Study (CHARLS) and the Health and Retirement Study (HRS). Initiated in 2011, CHARLS is an ongoing, nationally representative longitudinal survey of Chinese adults aged ≥ 45 years, employing multistage stratified probability‐proportionate‐to‐size sampling across 450 villages in 28 provinces, with over 17,000 baseline participants (Zhao et al. 2014). Data are collected via structured interviews by Peking University staff, with five waves released to date (2011–2020; Wave 1: 2011–2012, Wave 2: 2013–2014, Wave 3: 2015–2016, Wave 4: 2018, Wave 5: 2020). Ethical approval was granted by Peking University's Biomedical Ethics Review Committee (IRB00001052‐11015), with informed consent obtained from all participants (Zhao et al. 2014); more details regarding how CHARLS was designed, how the interviews were conducted, and what was the meaning for each item in the questionnaire can be found at http://CHARLS.pku.edu.cn/en.

As a parallel study of CHARLS, HRS is also a nationally representative longitudinal survey of more than 37,000 individuals ≥ 50 years old in 23,000 households in the United States (https://hrs.isr.umich.edu) (Sonnega et al. 2014). The survey was fielded every 2 years since 1992 in order to provide a national resource for data on the changing health and economic circumstances associated with aging at both the individual and population levels (Sonnega et al. 2014). Since 2006, HRS has incorporated biomarkers and genetic data, with recent waves (2016–2021) enhancing its utility for aging research. HRS ethics approval was secured from its survey committee, with participant consent obtained. For this cross‐sectional study, we analyzed Wave 3 (2015–2016) of CHARLS and Wave 14 (2018–2019) of HRS to provide current insights into sarcopenia status.

### 2.2 Data Imputation and Selection Criteria

Missing data in the CHARLS and HRS datasets were addressed using the Python miceforest package. This tool implements multiple imputation by chained equations (MICE) integrated with random forest models to predict and impute missing values, effectively capturing complex, nonlinear relationships (https://github.com/AnotherSamWilson/miceforest). The imputation process entailed initializing missing values, performing multiple iterations to refine imputations, and generating multiple imputed datasets to account for uncertainty. Specifically, 10 imputed datasets were created, each undergoing 10 iterations, to minimize randomness and enhance algorithmic accuracy. Imputation was restricted to variables with less than 80% missing values. This approach provided robustness to nonlinear data patterns, flexibility in processing mixed data types, and the ability to quantify imputation uncertainty through multiple imputations. Following imputation, participants aged under 50 years or dead were excluded, and the remaining individuals were included in formal analyses.

### 2.3 Diagnosis and Classification for Stroke Status

Stroke was the major exposure in the current study, and the diagnosis was made mainly based on self‐reported outcomes where individuals had received a confirmed diagnosis of stroke from a physician (Zheng et al. 2019; Li et al. 2019). The stroke onset period was defined as being between the most recent interview and the specific one when the stroke incidence was reported by participants. Take CHARLS as an example, the participants would be asked the following critical question: Have you ever been diagnosed with a stroke by a physician? Since stroke shares overlaps with CVD, in line with previous studies (Zheng et al. 2019; Xie et al. 2019), CVD was assessed by the critical question—Have you ever been diagnosed with a heart attack, coronary heart disease, angina, congestive heart failure or other heart problems by a physician? or Have you ever been diagnosed with a stroke by a physician?—to ascertain these covariables. Participants who reported stroke with or without other CVDs were considered as stroke patients, and participants who reported suffering CVD but without stroke were not considered. Due to limitations of the self‐reported survey and questionnaires, we could not define the detailed subtypes of stroke, so all stroke types were analyzed together in the current study.

### 2.4 Diagnosis and Classification for Sarcopenia Status

Given the racial differences of participants in CHARLS and HRS, we applied updated AWGS2019 (L.‐K. Chen et al. 2020) and EWGSOP2 (Cruz‐Jentoft et al. 2019) for sarcopenia diagnosis, respectively. AWGS2019 involved three modules: muscle strength, muscle mass, and physical performance (L.‐K. Chen et al. 2020). Sarcopenia was identified when low muscle mass co‐occurred with either low muscle strength or low physical performance. Possible sarcopenia was defined with the presence of low muscle strength or low physical performance without fulfilling the full sarcopenia criteria (L.‐K. Chen et al. 2020). Severe sarcopenia was classified when low muscle mass, low muscle strength, and low physical performance were all present. Participants not meeting these conditions were categorized as no sarcopenia (L.‐K. Chen et al. 2020). Muscle strength was measured via grip strength, recorded twice by trained examiners in CHARLS, with the average of the maximum values adopted; cutoff thresholds were < 18 kg for female and < 28 kg for male (L.‐K. Chen et al. 2020). Physical performance was assessed using gait speed, the five‐repetition chair stand test, and the short physical performance battery (SPPB), with low performance defined as gait speed < 1.0 m/s, chair stand test ≥ 12 s, or SPPB score ≤ 9 (L.‐K. Chen et al. 2020). Muscle mass was evaluated with appendicular skeletal muscle (ASM) mass calculated by a previously validated and widely used anthropometric equation, and consistency between ASM derived from this equation and dual‐energy x‐ray absorptiometry (DXA) has been proven (Hu et al. 2022; Wen et al. 2011).

$$\begin{array}{c} \mathrm{ASM}=0.193\times \mathrm{weight}\left(\mathrm{kg}\right)+0.107\times \mathrm{height}\left(\mathrm{cm}\right)-4.157\times \mathrm{sex}\\ \left(1\mathrm{for}\mathrm{male},2\mathrm{for}\mathrm{female}\right)-0.037\times \mathrm{age}\left(\mathrm{year}\right)-2.631 \end{array}$$

Height and weight were measured per survey protocols, and height‐adjusted muscle mass (ASM/Ht^2^) was derived by dividing ASM by height squared. Low muscle mass was defined as ASM/Ht^2^ < 4.90 kg/m^2^ for female and < 6.79 kg/m^2^ for male (Hu et al. 2022; Wen et al. 2011).

In HRS, due to the absence of chair stand test data, physical performance was evaluated solely by gait speed. According to EWGSOP2, possible sarcopenia was defined as low muscle strength without low muscle mass, sarcopenia as low muscle strength plus low muscle mass without low physical performance, and severe sarcopenia as the concurrence of low muscle strength, low muscle mass, and low physical performance. Participants lacking low muscle strength and not meeting possible sarcopenia, sarcopenia, or severe sarcopenia criteria were classified as no sarcopenia (Cruz‐Jentoft et al. 2019). Cutoff values were < 27 kg for male and < 16 kg for female for low muscle strength, ASM/Ht^2^ < 7.0 kg/m^2^ for male and < 5.5 kg/m^2^ for female for low muscle mass (without height adjustment), and gait speed < 0.8 m/s for low physical performance (Cruz‐Jentoft et al. 2019).

### 2.5 Assessment of Stroke and Covariables

Stroke was evaluated based on participants’ self‐reported responses to questionnaire items, consistent with previous studies (Gao et al. 2022; Bing et al. 2025). Covariates included sociodemographic factors—age, gender, education, marital status, and residence—and health‐related factors: smoking, alcohol consumption, eating disorders, dyslipidemia, liver disease, kidney disease, digestive disease, psychiatric disorders, cognitive impairment, hypertension, diabetes, metabolic syndrome (MetS), C‐reactive protein (CRP), HbA1c, body mass index (BMI), medication use on diabetes, digestive disease, dyslipidemia and hypertension, cancer, obesity, fatigue, angina, congestive heart failure, arrhythmia, instrumental activities of daily living (IADL) score, daily cigarette consumption, and weekly exercise frequency. Due to variations in variable availability, the covariates adjusted in CHARLS and HRS differed slightly.

In CHARLS, the IADL scale comprised six items: household chores, preparing hot meals, grocery shopping, managing finances, medication adherence, and phone use, each scored as 0 (*no difficulty*) or 1 (*difficulty*), yielding a range from 0 (*fully independent*) to 6 (highly dependent) (Hopkins et al. 2017). In HRS, the IADL scale included five items, excluding household chores, with scores ranging from 0 to 5, where higher scores indicated greater functional impairment (Hopkins et al. 2017). MetS was assessed using standardized criteria, such as the National Cholesterol Education Program Adult Treatment Panel III (NCEP/ATP III) or International Diabetes Federation (IDF) definitions, integrating self‐reported and clinical data (Fahed et al. 2022). Physician‐diagnosed covariates were first considered; in their absence, self‐reported data were utilized, with quantitative measures derived from survey‐specific parameters.

### 2.6 Statistical Analysis

Descriptive statistics were reported as means ± standard deviations (SDs) or medians with interquartile ranges (IQRs) for continuous variables, and as frequencies with percentages for categorical variables. Group differences were analyzed using chi‐square tests (or Fisher's exact test) for categorical variables and one‐way ANOVA or the Kruskal–Wallis *H* test for continuous variables, as appropriate.

Logistic regression models were used to estimate odds ratios (ORs) with 95% confidence intervals (CIs) for the associations between stroke and various sarcopenia statuses and their components in the main cross‐sectional analyses, with adjustments applied across multiple models and subgroup analyses. Multicollinearity among covariates was assessed using the variance inflation factor (VIF), with VIF > 5 indicating significant multicollinearity and exclusion from the model. Stepwise regression was employed to select a subset of independent variables, optimizing model fit and convergence by iteratively adding or removing variables based on statistical criteria. It combined forward selection (adding variables) and backward elimination (removing variables) to optimize a predefined criterion, and variables contributing little explanatory power would be excluded to reduce overfitting and multicollinearity. Mediation effects of key variables (eating disorders, CRP, IADL, and BMI) that were interesting and exhibited strong explanatory values were examined using the Baron and Kenny (1986) method after stepwise regression analysis. The relationship between CRP and sarcopenia status was further analyzed using continuous‐scale restricted cubic splines (RCSs) with four knots within a logistic regression framework based on the “rcssci” R package. Four knots were taken to best fit the RCS just like previous studies, and splines were adjusted for stroke. The cutoff points were determined based on a likelihood‐ratio test using the “segmented” R package.

Sensitivity analyses utilized generalized estimating equation (GEE) models to cross‐validate results from the logistic regression model. Also, HRS findings could be treated as an additional sensitivity analysis to reinforce CHARLS outcomes. This study adhered to the Strengthening the Reporting of Observational Studies in Epidemiology (STROBE) guidelines (**Table** **S1**) (von Elm et al. 2007). All statistical analyses were performed using Python 3.9 (https://www.python.org/) and R 4.3.0 (https://www.r‐project.org/), and used packages are addressed throughout the whole article, with statistical significance set at a two‐sided *p*‐value < 0.05.

## 3 Results

### 3.1 Baseline Characteristics of the Study Populations

**FIGURE 1:**
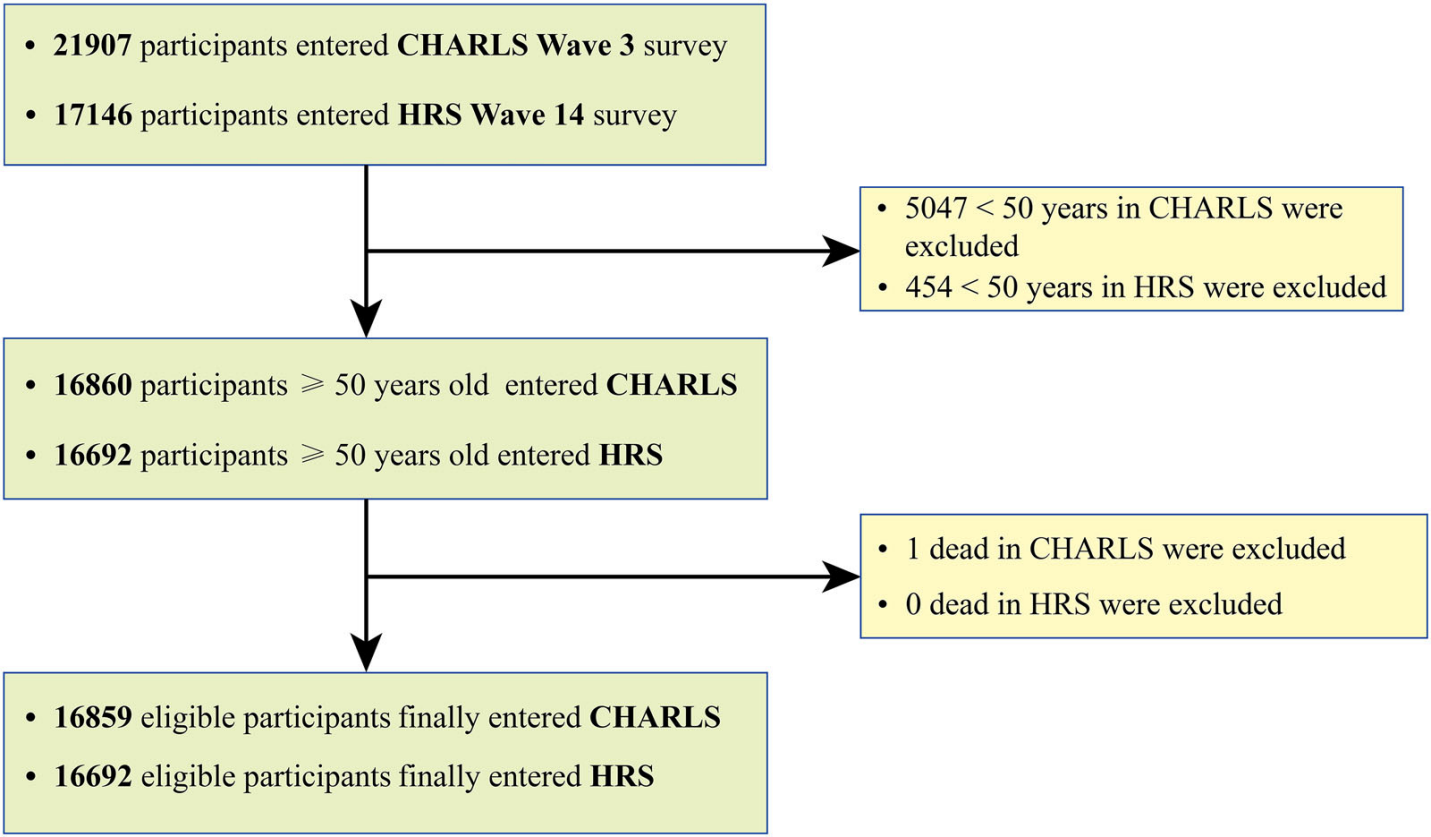
The flow chart of participants selection.

Following the inclusion criteria (Figure 1), the study included 16,859 participants from the CHARLS cohort (51% female, mean age: 62.90 ± 9.17 years) and 16,692 participants from the HRS cohort (59% female, mean age: 67.60 ± 10.89 years). Detailed baseline characteristics are presented in Tables 1 and 2. In the CHARLS cohort, the distribution of sarcopenia statuses was as follows: 7.6% no sarcopenia, 83.2% possible sarcopenia, 5.3% sarcopenia, and 3.9% severe sarcopenia. As sarcopenia severity increased, participants exhibited higher frailty indices and greater prevalences of digestive diseases (severe sarcopenia: 39%), eating disorders (severe sarcopenia: 8.3%), stroke medication use (severe sarcopenia: 7.7%), digestive disease medication use (severe sarcopenia: 31%), psychiatric disorders (severe sarcopenia: 4.2%), and cognitive impairments (severe sarcopenia: 29%), alongside lower BMI and reduced dyslipidemia rates (severe sarcopenia: 14%). Regarding stroke, 94.8% of participants reported no stroke history, while 5.2% had experienced a stroke. Compared to those without stroke, stroke patients demonstrated significantly higher rates of comorbidities, including non‐stroke CVDs (47%), liver disease (15%), kidney disease (23%), MetS (79%), and psychiatric disorders (7.8%), as well as increased medication use (e.g., 41% for heart disease). Additionally, stroke patients exhibited elevated CRP levels, diminished self‐care abilities, and reduced physical activity (lower total metabolic equivalent of task (MET)) (Table S2).

**TABLE 1:** Sarcopenia‐based patient characteristics for CHARLS.

	Overall (*n* = 16,859)	No sarcopenia (*n* = 1289)	Possible sarcopenia (*n* = 14,023)	Sarcopenia (*n* = 886)	Severe sarcopenia (*n* = 661)	*p*‐value
Age (year)	62.90± (9.17)	63.08± (6.50)	61.92± (8.85)	69.32± (8.65)	74.73± (9.18)	< 0.001
Low muscle mass	1656 (9.8%)	109 (8.5%)	0 (0%)	886 (100%)	661 (100%)	< 0.001
Low muscle strength	2294 (14%)	0 (0%)	1607 (11%)	26 (2.9%)	661 (100%)	< 0.001
Low physical performance	15,478 (92%)	0 (0%)	13,957 (100%)	860 (97%)	661 (100%)	< 0.001
Chair stand test (s)	10.02± (3.87)	7.69± (2.18)	10.04± (3.70)	10.32± (3.88)	13.76± (6.19)	< 0.001
Frailty index	18.44± (12.59)	14.35± (9.33)	18.32± (12.52)	19.52± (12.42)	27.42± (15.05)	< 0.001
Left hand strength (kg)	27.68± (9.84)	31.27± (9.16)	28.10± (9.70)	24.87± (7.61)	15.53± (6.75)	< 0.001
Right hand strength (kg)	29.06± (10.23)	32.81± (9.51)	29.50± (10.09)	26.20± (8.00)	16.26± (6.68)	< 0.001
BMI (kg/m^2^)	24.59± (23.14)	24.40± (13.34)	24.71± (12.88)	25.41± (82.54)	21.36± (25.74)	< 0.001
Total MET (MET—h/week)^a^	6340.00± (5153.70)	8185.66± (5982.92)	6220.22± (5014.53)	6802.84± (5446.27)	4661.48± (4949.77)	< 0.001
Height (m)	1.58± (0.09)	1.59± (0.11)	1.58± (0.08)	1.48± (0.15)	1.48± (0.11)	< 0.001
Waist (cm)	85.48± (11.97)	84.46± (12.37)	86.88± (10.99)	71.90± (14.63)	75.96± (11.39)	< 0.001
Weight (kg)	59.41± (10.97)	60.03± (11.59)	61.09± (9.95)	44.03± (5.41)	43.35± (5.65)	< 0.001
Systolic pressure (mmHg)	128.54± (18.72)	127.88± (17.92)	128.54± (18.33)	127.66± (22.06)	130.98± (22.98)	0.002
TYG	8.72± (0.54)	8.68± (0.61)	8.75± (0.53)	8.45± (0.49)	8.48± (0.52)	< 0.001
TYG BMI	213.34± (120.91)	211.79± (113.28)	216.35± (113.59)	194.93± (216.62)	177.18± (96.86)	< 0.001
Total cholesterol (mg/dL)	185.04± (31.01)	186.08± (36.06)	185.19± (30.26)	183.96± (32.45)	181.44± (33.79)	0.030
TG (mg/dL)	140.46± (74.41)	138.55± (85.17)	144.04± (74.25)	109.31± (59.72)	110.03± (53.35)	< 0.001
HDL cholesterol (mg/dL)	51.44± (9.89)	52.16± (11.16)	50.83± (9.29)	56.99± (11.86)	55.44± (12.96)	< 0.001
LDL cholesterol (mg/dL)	103.45± (24.61)	104.05± (27.65)	103.60± (24.10)	102.03± (26.30)	101.02± (26.45)	0.014
CRP	2.86± (5.20)	2.22± (3.40)	2.81± (4.80)	3.29± (8.23)	4.63± (9.19)	< 0.001
HbA1c (%)	6.01± (0.87)	6.00± (0.93)	6.03± (0.88)	5.86± (0.63)	5.97± (0.95)	< 0.001
Gender						< 0.001
Female	8597 (51%)	545 (42%)	7269 (52%)	457 (52%)	326 (49%)	
Male	8262 (49%)	744 (58%)	6754 (48%)	429 (48%)	335 (51%)	
Hypertension	6260 (37%)	430 (33%)	5367 (38%)	238 (27%)	225 (34%)	< 0.001
Dyslipidemia	4143 (25%)	334 (26%)	3611 (26%)	105 (12%)	93 (14%)	< 0.001
Diabetes	2673 (16%)	189 (15%)	2331 (17%)	79 (8.9%)	74 (11%)	< 0.001
Liver disease^b^	1183 (7.0%)	96 (7.4%)	997 (7.1%)	51 (5.8%)	39 (5.9%)	0.260
Heart disease	4104 (24%)	294 (23%)	3506 (25%)	151 (17%)	153 (23%)	< 0.001
Kidney disease^b^	1778 (11%)	122 (9.5%)	1483 (11%)	96 (11%)	77 (12%)	0.470
Digestive disease	5655 (34%)	424 (33%)	4629 (33%)	345 (39%)	257 (39%)	< 0.001
Psychiatric disease	413 (2.4%)	29 (2.2%)	328 (2.3%)	28 (3.2%)	28 (4.2%)	0.009
Cognitive status	4156 (25%)	276 (21%)	3424 (24%)	264 (30%)	192 (29%)	< 0.001
Headache	2514 (15%)	120 (9.3%)	2079 (15%)	170 (19%)	145 (22%)	< 0.001
Eating disorders	521 (3.1%)	9 (0.7%)	429 (3.1%)	28 (3.2%)	55 (8.3%)	< 0.001
Rural living	10,043 (60%)	737 (57%)	8132 (58%)	663 (75%)	511 (77%)	< 0.001
Married						< 0.001
Others	2612 (15%)	149 (12%)	1986 (14%)	234 (26%)	243 (37%)	
Yes	14,247 (85%)	1140 (88%)	12,037 (86%)	652 (74%)	418 (63%)	
Drinking	7822 (46%)	683 (53%)	6425 (46%)	407 (46%)	307 (46%)	< 0.001
Smoking	7651 (45%)	664 (52%)	6211 (44%)	434 (49%)	342 (52%)	< 0.001
Education						< 0.001
Below high school	14,787 (88%)	1113 (86%)	12,167 (87%)	862 (97%)	645 (98%)	
College/university	333 (2.0%)	26 (2.0%)	304 (2.2%)	3 (0.3%)	0 (0%)	
High school	1739 (10%)	150 (12%)	1552 (11%)	21 (2.4%)	16 (2.4%)	
MetS	9960 (59%)	661 (51%)	8660 (62%)	321 (36%)	318 (48%)	< 0.001
Diabetes medication use	2445 (15%)	171 (13%)	2134 (15%)	72 (8.1%)	68 (10%)	< 0.001
Digestive medication use	4149 (25%)	296 (23%)	3383 (24%)	262 (30%)	208 (31%)	< 0.001
Dyslipidemia medication use	2875 (17%)	208 (16%)	2516 (18%)	76 (8.6%)	75 (11%)	< 0.001
Heart disease medication use	3439 (20%)	239 (19%)	2938 (21%)	120 (14%)	142 (21%)	< 0.001
Hypertension medication use	5268 (31%)	339 (26%)	4549 (32%)	193 (22%)	187 (28%)	< 0.001
Stroke medication use	766 (4.5%)	27 (2.1%)	652 (4.6%)	36 (4.1%)	51 (7.7%)	< 0.001

*Note*: Data were displayed in mean ± standard deviation, median (interquartile range), or number (percentages).

Abbreviations: BMI, body mass index; CRP, C‐reactive protein; HDL, high‐density lipoprotein cholesterol; LDL, low‐density lipoprotein cholesterol; MET, metabolic equivalent of task; MetS, metabolism syndrome; TG, triglyceride; TYG, triglyceride‐glucose index.

^a^ Evaluated for total metabolic equivalent of task, 1 MET = 1 kcal/kg × h.

^b^ Indicate chronic diseases.

**TABLE 2:** Sarcopenia‐based patient characteristics for HRS.

	Overall (*n* = 16,692)	No sarcopenia (*n* = 14,512)	Possible sarcopenia (*n* = 2058)	Sarcopenia (*n* = 18)	Severe sarcopenia (*n* = 104)	*p*‐value
Age	67.60± (10.89)	65.89± (9.87)	78.89± (10.54)	75.89± (10.89)	81.38± (10.19)	< 0.001
Low muscle mass	331 (2.0%)	209 (1.4%)	0 (0%)	18 (100%)	104 (100%)	< 0.001
Low muscle strength	2180 (13%)	0 (0%)	2058 (100%)	18 (100%)	104 (100%)	< 0.001
Low physical performance	12,246 (73%)	10,332 (71%)	1810 (88%)	0 (0%)	104 (100%)	< 0.001
Education year	12.86± (3.25)	12.98± (3.18)	12.00± (3.62)	14.44± (2.25)	12.38± (3.34)	< 0.001
ADL	0.47± (1.18)	0.35± (0.98)	1.32± (1.91)	0.17± (0.51)	0.70± (1.28)	< 0.001
Cigarette (number/day)	1.44± (4.67)	1.52± (4.80)	0.78± (3.56)	2.78± (6.69)	1.72± (4.90)	< 0.001
Exercise frequency (times/week)	4.43± (1.66)	4.31± (1.61)	5.26± (1.74)	3.72± (2.03)	4.96± (1.97)	< 0.001
Left hand strength (kg)	27.24± (10.55)	28.72± (10.31)	17.62± (5.76)	14.99± (3.23)	13.88± (3.99)	< 0.001
Right hand strength (kg)	32.69± (9.98)	33.74± (9.74)	25.91± (8.77)	22.93± (6.96)	21.96± (6.20)	< 0.001
BMI (kg/m^2^)	30.50± (4.09)	30.58± (4.00)	30.55± (4.13)	20.16± (2.05)	20.69± (2.49)	< 0.001
Height (m)	1.65± (0.10)	1.66± (0.10)	1.61± (0.11)	1.57± (0.09)	1.56± (0.10)	< 0.001
Weight (kg)	83.78± (14.46)	84.65± (14.13)	79.58± (14.02)	49.40± (5.52)	50.28± (6.34)	< 0.001
Waist (cm)	41.47± (5.45)	41.46± (5.44)	42.10± (5.13)	31.17± (3.47)	32.54± (3.22)	< 0.001
IADL	0.35± (0.95)	0.25± (0.75)	1.08± (1.61)	0.28± (0.75)	0.60± (1.16)	< 0.001
Systolic pressure (mmHg)	127.33± (13.53)	127.22± (13.22)	128.10± (15.16)	123.67± (17.67)	128.25± (19.55)	0.002
Diastolic pressure (mmHg)	77.19± (8.26)	77.78± (8.01)	73.23± (8.61)	74.72± (12.31)	73.62± (11.90)	< 0.001
speed (m/s)	0.77± (0.51)	0.79± (0.50)	0.63± (0.57)	1.10± (0.56)	0.52± (0.18)	< 0.001
Married						< 0.001
Others	7619 (46%)	6369 (44%)	1166 (57%)	8 (44%)	76 (73%)	
Yes	9073 (54%)	8143 (56%)	892 (43%)	10 (56%)	28 (27%)	
Gender						< 0.001
Female	9765 (59%)	8466 (58%)	1195 (58%)	15 (83%)	89 (86%)	
Male	6927 (41%)	6046 (42%)	863 (42%)	3 (17%)	15 (14%)	
Rural living						< 0.001
No	9189 (55%)	8132 (56%)	999 (49%)	10 (56%)	48 (46%)	
Others	3598 (22%)	3055 (21%)	513 (25%)	3 (17%)	27 (26%)	
Yes	3905 (23%)	3325 (23%)	546 (27%)	5 (28%)	29 (28%)	
Smoking	8996 (54%)	7856 (54%)	1079 (52%)	7 (39%)	54 (52%)	0.27
Drinking	9358 (56%)	8482 (58%)	823 (40%)	8 (44%)	45 (43%)	< 0.001
Hypertension	10,540 (63%)	8879 (61%)	1577 (77%)	10 (56%)	74 (71%)	< 0.001
Diabetes	4822 (29%)	4061 (28%)	733 (36%)	4 (22%)	24 (23%)	< 0.001
Cancer	2578 (15%)	2064 (14%)	488 (24%)	8 (44%)	18 (17%)	< 0.001
Heart disease	4173 (25%)	3253 (22%)	871 (42%)	7 (39%)	42 (40%)	< 0.001
Psychiatric disease	3030 (18%)	2638 (18%)	376 (18%)	3 (17%)	13 (13%)	0.52
Headache	1396 (8.4%)	1074 (7.4%)	305 (15%)	5 (28%)	12 (12%)	< 0.001
Fatigue	3046 (18%)	2264 (16%)	746 (36%)	5 (28%)	31 (30%)	< 0.001
Angina	1486 (8.9%)	1167 (8.0%)	302 (15%)	3 (17%)	14 (13%)	< 0.001
Congestive heart failure	1258 (7.5%)	930 (6.4%)	311 (15%)	2 (11%)	15 (14%)	< 0.001
Arrhythmia	2824 (17%)	2179 (15%)	611 (30%)	3 (17%)	31 (30%)	< 0.001
Dyslipidemia	9862 (59%)	8498 (59%)	1294 (63%)	10 (56%)	60 (58%)	0.003
Hypertension medication use	9202 (55%)	7701 (53%)	1427 (69%)	9 (50%)	65 (63%)	< 0.001
Diabetes medication use	3468 (21%)	2916 (20%)	532 (26%)	3 (17%)	17 (16%)	< 0.001
Stroke medication use	493 (3.0%)	351 (2.4%)	134 (6.5%)	2 (11%)	6 (5.8%)	< 0.001
Angina medication use	991 (5.9%)	742 (5.1%)	238 (12%)	3 (17%)	8 (7.7%)	< 0.001
Congestive heart failure medication use	832 (5.0%)	598 (4.1%)	221 (11%)	1 (5.6%)	12 (12%)	< 0.001
Psychiatric medication use	1787 (11%)	1498 (10%)	278 (14%)	2 (11%)	9 (8.7%)	< 0.001
Heart disease medication use	306 (1.8%)	230 (1.6%)	70 (3.4%)	1 (5.6%)	5 (4.8%)	< 0.001
Dyslipidemia medication use	7231 (43%)	6072 (42%)	1105 (54%)	9 (50%)	45 (43%)	< 0.001
Digestive medication use	2893 (17%)	2338 (16%)	532 (26%)	3 (17%)	20 (19%)	< 0.001
Physical activity use	6506 (39%)	5957 (41%)	505 (25%)	10 (56%)	34 (33%)	< 0.001
Obesity	7445 (45%)	6532 (45%)	912 (44%)	0 (0%)	1 (1.0%)	< 0.001

Abbreviations: ADL, activities of daily living; BMI, body mass index; IADL, instrumental activities of daily living.

In the HRS cohort, the sarcopenia status distribution was 86.9% no sarcopenia, 12.3% possible sarcopenia, 0.1% sarcopenia, and 0.6% severe sarcopenia (Table 2), with 90.7% reporting no stroke and 9.3% reporting a stroke (Table S3). Demographic characteristics across SA and stroke statuses were generally consistent with those observed in CHARLS. Notably, most Chinese participants were classified as possible sarcopenia followed by no sarcopenia, whereas most US participants were no sarcopenia, likely attributable to differences in diagnostic criteria (AWGS2019 vs. EWGSOP2), ethnic variations, socioeconomic factors, and social structures. We used advanced artificial intelligence (AI)‐based imputation to preserve the original data structure, yielding comparable combined no sarcopenia and possible sarcopenia proportions across cohorts (90.8% in CHARLS and 99.2% in HRS). Regarding the stroke prevalence, the US populations seemed to exhibit higher rates than the Chinese populations, which could be because we set age selection criteria (≥ 50 years), and the mean age was slightly higher in HRS.

### 3.2 Association Between Stroke and Sarcopenia Events in CHARLS

In the unadjusted logistic regression model, stroke was associated with a 107% increased risk of incident possible sarcopenia compared to no stroke (OR: 2.07, 95% CI: 1.49–2.99). This association remained significant across all adjusted models: Model 1 (OR: 2.28, 95% CI: 1.63–3.30), Model 2 (OR: 1.95, 95% CI: 1.38–2.83), Model 3 (OR: 1.89, 95% CI: 1.33–2.77), and Model 4 (OR: 1.86, 95% CI: 1.31–2.73). For sarcopenia incidence, stroke was linked to a significantly elevated risk in the unadjusted model (OR: 1.93, 95% CI: 1.23–3.06), with consistent findings in adjusted Models 1 (OR: 1.72, 95% CI: 1.03–2.89), 2 (OR: 1.86, 95% CI: 1.07–3.27), 3 (OR: 1.90, 95% CI: 1.08–3.34), and 4 (OR: 1.86, 95% CI: 1.06–3.27). For severe sarcopenia, stroke was associated with a significantly increased risk in the unadjusted model (OR: 3.42, 95% CI: 2.22–5.34), Model 1 (OR: 2.57, 95% CI: 1.20–5.56), and Model 2 (OR: 2.48, 95% CI: 1.09–5.76). Although not statistically significant in Models 3 and 4, a strong trend toward increased severe sarcopenia risk persisted. Detailed results and covariates for each model are presented in Table 3. No multicollinearity was detected among covariates using the VIF tool (all VIF < 5).

**TABLE 3:** Unadjusted and adjusted association between stroke and risk for different sarcopenia status in CHARLS and HRS.

	CHARLS cohort results										
vs. no sarcopenia	Event/total individuals	Unadjusted model		Model 1^a^		Model 2^b^		Model 3^c^		Model 4^d^	
Possible sarcopenia	14,023/15,312	OR (95% CI)	*p*‐value	OR (95% CI)	*p*‐value	OR (95% CI)	*p*‐value	OR (95% CI)	*p*‐value	OR (95% CI)	*p*‐value
No stroke	13,278/14,533	1 (reference)		1 (reference)		1 (reference)		1 (reference)		1 (reference)	
Stroke	745/779	2.07 (1.49–2.99)	< 0.001	2.28 (1.63–3.30)	< 0.001	1.95 (1.38–2.83)	< 0.001	1.89 (1.33–2.77)	< 0.001	1.86 (1.31–2.73)	< 0.001
Sarcopenia	886/2175										
No stroke	842/2097	1 (reference)		1 (reference)		1 (reference)		1 (reference)		1 (reference)	
Stroke	44/78	1.93 (1.23–3.06)	0.005	1.72 (1.03–2.89)	0.038	1.86 (1.07–3.27)	0.029	1.90 (1.08–3.34)	0.025	1.86 (1.06–3.27)	0.031
Severe sarcopenia	661/1950										
No stroke	605/1860	1 (reference)		1 (reference)		1 (reference)		1 (reference)		1 (reference)	
Stroke	56/90	3.42 (2.22–5.34)	< 0.001	2.57 (1.20–5.56)	0.016	2.48 (1.09–5.76)	0.033	2.29 (1.00–5.31)	0.053	2.22 (0.97–5.19)	0·062
	**HRS cohort results**										
**vs. no sarcopenia**	**Event/Total individuals**	**Unadjusted model**		**Model 1^e^**		**Model 2^f^**		**Model 3^g^**		**Model 4^h^**	
Possible sarcopenia	2058/16,570	OR (95% CI)	*p*‐value	OR (95% CI)	*p*‐value	OR (95% CI)	*p*‐value	OR (95% CI)	*p*‐value	OR (95% CI)	*p*‐value
No stroke	1637/15,041	1 (reference)		1 (reference)		1 (reference)		1 (reference)		1 (reference)	
Stroke	421/1529	3.11 (2.75–3.52)	< 0.001	2.01 (1.73–2.33)	< 0.001	1.33 (1.13–1.56)	< 0.001	1.23 (1.04–1.45)	0.014	1.22 (1.04–1.44)	0.017
Sarcopenia	18/14,530										
No stroke	15/13,419	1 (reference)		1 (reference)		1 (reference)		1 (reference)		1 (reference)	
Stroke	3/1111	2.42 (0.56–7.35)	0·163	1.71 (0.27–6.53)	0·491	3.18 (1.49–6.53)	< 0.002	3.85 (2.12–6.83)	< 0.001	3.82 (2.02–7.10)	< 0.001
Severe sarcopenia	104/14,616										
No stroke	88/13,492	1 (reference)		1 (reference)		1 (reference)		1 (reference)		1 (reference)	
Stroke	16/1124	2.20 (1.24–3.65)	0.004	1.27 (0.61–2.41)	0.485	1.23 (0.94–1.61)	0.129	1.30 (0.99–1.69)	0.055	1.27 (0.96–1.66)	0.085

*Note*: For CHARLS results, ^a^Model 1 was adjusted for age and gender; ^b^Model 2 was adjusted for Model 1 plus BMI, marital status, residence, education, smoking, drinking, MetS, eating difficulties, dyslipidemia, liver disease, kidney disease, digestive disease, psychiatric disease, and cognitive status; ^c^Model 3 was adjusted for Model 2 plus systolic, CRP, HbA1c, hypertension, and diabetes; and ^d^Model 4 was adjusted for Model 3 plus diabetes medication use, digestive medication use, dyslipidemia medication use, and hypertension medication use. For HRS results, ^e^Model 1 was adjusted for age and gender; ^f^Model 2 was adjusted for Model 1 plus BMI, marital status, education year, smoking, drinking, dyslipidemia, psychiatric disease, cancer, obesity, fatigue, angina, congestive heart failure, and arrhythmia; ^g^Model 3 was adjusted for Model 2 plus IADL, cigarette consumption, and exercise frequency; and ^h^Model 4 was adjusted for Model 3 plus diabetes, diabetes medication use, dyslipidemia medication use, and hypertension medication use.

Abbreviations: OR, odds ratio; 95% CI, 95% confidence interval.

**FIGURE 2:**
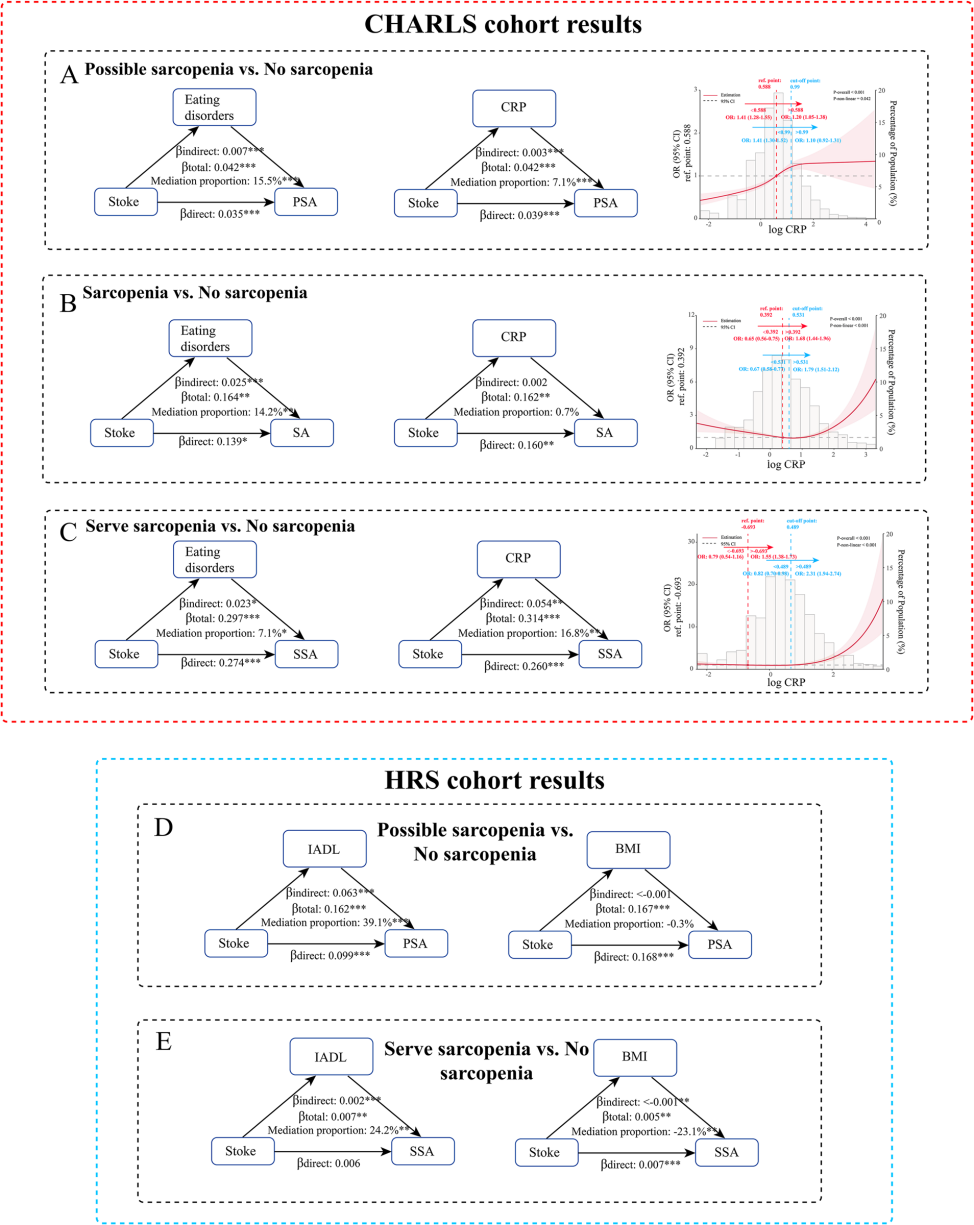
Mediation Analysis and RCS curves of Key Factors in the Stroke‐Sarcopenia Association. In CHARLS, path diagram of the mediated relationship of eating disorders between stroke and possible sarcopenia, and mediated relationship of CRP between stroke and possible sarcopenia were displayed in A; the mediated role of eating disorders and CRP between stroke and sarcopenia were displayed in B; the mediated role of eating disorders and CRP between stroke and severe sarcopenia were displayed in C. In HRS, the mediated role of IADL and BMI between stroke and possible sarcopenia were displayed in D; the mediated role of IADL and BMI between stroke and severe sarcopenia were displayed in E. RCS curve showed dose‐response relationship between log CRP and possible sarcopenia (A), sarcopenia (B) and severe sarcopenia (C) in which reference point as well as cut‐off point was marked, also the ORs with 95% CIs showing trends in both sides of these points were shown. From the RCS, we could observe the changes of risk on sarcopenia status and their trends when log CRP was below and above the cut‐off point. *p value < 0.05, **p value < 0.01, **p value < 0.001. Abbreviations: CRP, C‐reactive protein; PSA, possible sarcopenia, SA, sarcopenia, SSA, serve sarcopenia; IADL, instrumental activities of daily living; BMI, body mass index; OR, odd ratio; CI, confidence interval.

Following stepwise regression analysis to quantify associations between covariates and SA status (Table S4), the mediation effects of eating disorders and CRP were examined. Eating disorders significantly mediated the relationship between stroke and possible sarcopenia (15.5% of the total effect), stroke and sarcopenia (14.2%), and stroke and severe sarcopenia (7.1%) (Figure 2). Similarly, CRP mediated the stroke–possible sarcopenia and stroke–severe sarcopenia relationships, with higher CRP levels accounting for 7.1% and 16.8% of the total effects, respectively. However, CRP did not significantly mediate the stroke–sarcopenia relationship due to a low indirect effect (Figure 2). These findings suggested that systemic inflammation, as indicated by CRP, played an increasingly prominent role in mediating the stroke–sarcopenia association as sarcopenia severity increased, whereas the mediating effect of eating disorders diminished.

The dose–response relationship between CRP and possible sarcopenia risk was visualized using RCS curves (Figure 2A). Both overall and nonlinear associations were significant (P for nonlinearity = 0.042). A cutoff point at log CRP = 0.99 divided the plot into two phases: for log CRP < 0.99, possible sarcopenia risk increased rapidly (OR: 1.41, 95% CI: 1.30–1.52; cutoff point log CRP = 0.99 vs. the starting point of Plot Phase 1), while for log CRP > 0.99, the risk plateaued (OR: 1.10, 95% CI: 0.92–1.31; the end point of Plot Phase 2 vs. cutoff point). The reference point (OR: 1.0) was at log CRP = 0.588, with ORs remaining above 1 for log CRP > 0.588. For sarcopenia, the RCS curve (Figure 2B) identified a cutoff at log CRP = 0.531 delineating the plot into two phases, with sarcopenia risk decreasing steadily for log CRP < 0.531 (OR: 0.67, 95% CI: 0.58–0.77; cutoff point vs. starting point of Plot Phase 1) and increasing gradually for log CRP > 0.531 (OR: 1.79, 95% CI: 1.51–2.12; the end point of Plot Phase 2 vs. cutoff point). Notably, ORs remained below 1 between log CRP = 0.392 and 0.531. Both overall and nonlinear associations were significant (*p* for nonlinearity < 0.001). For severe sarcopenia, the RCS curve (Figure 2C) revealed a cutoff at log CRP = 0.489 separating the plots into two phases, with severe sarcopenia risk decreasing slightly for log CRP < 0.489 (OR: 0.82, 95% CI: 0.70–0.98) and increasing rapidly for log CRP > 0.489 (OR: 2.31, 95% CI: 1.94–2.74). Similar patterns were observed relative to the reference point at log CRP = −0.693, with significant overall and nonlinear associations (*p* for nonlinearity < 0.001).

### 3.3 Association Between Stroke and Sarcopenia Events in HRS

In the HRS cohort, findings were largely consistent with those from CHARLS. Stroke was associated with a 211% increased risk of possible sarcopenia compared to no stroke (OR: 3.11, 95% CI: 2.75–3.52). This association remained significant across all adjusted models: Model 1 (OR: 2.01, 95% CI: 1.73–2.33), Model 2 (OR: 1.33, 95% CI: 1.13–1.56), Model 3 (OR: 1.23, 95% CI: 1.04–1.45), and Model 4 (OR: 1.22, 95% CI: 1.04–1.44). For sarcopenia, no significant association was observed in the unadjusted model or Model 1, but significant increases were detected in Models 2 (OR: 3.18, 95% CI: 1.49–6.53), 3 (OR: 3.85, 95% CI: 2.12–6.83), and 4 (OR: 3.82, 95% CI: 2.02–7.10). For severe sarcopenia, stroke was significantly associated with increased risk in the unadjusted model (OR: 2.20, 95% CI: 1.24–3.65) but not in the adjusted models (Table 3). The lack of significance in some models may be attributable to the small number of sarcopenia and severe sarcopenia cases in HRS, which limited the statistical power of the logistic regression. No multicollinearity was detected among covariates.

Stepwise regression analysis of covariates is summarized in Table S4. Mediation effects of IADL and BMI on the stroke–sarcopenia relationship were explored. Due to the limited number of sarcopenia cases (*n* = 18), mediation effects for sarcopenia were not analyzed. IADL significantly mediated the stroke–possible sarcopenia and stroke–severe sarcopenia relationships, with higher IADL scores accounting for 39.1% and 24.2% of the total effects, respectively. Conversely, BMI appeared to exert a protective mediating effect, with higher BMI offsetting the risk for sarcopenia and severe sarcopenia by −0.3% and −23.1%, respectively (Figures 2D,E). These findings indicate that impaired self‐care ability in stroke patients substantially contributes to increased possible sarcopenia and severe sarcopenia risk, while higher BMI may serve as a protective factor, particularly for severe sarcopenia.

### 3.4 Subgroup and Sensitivity Analysis

**FIGURE 3:**
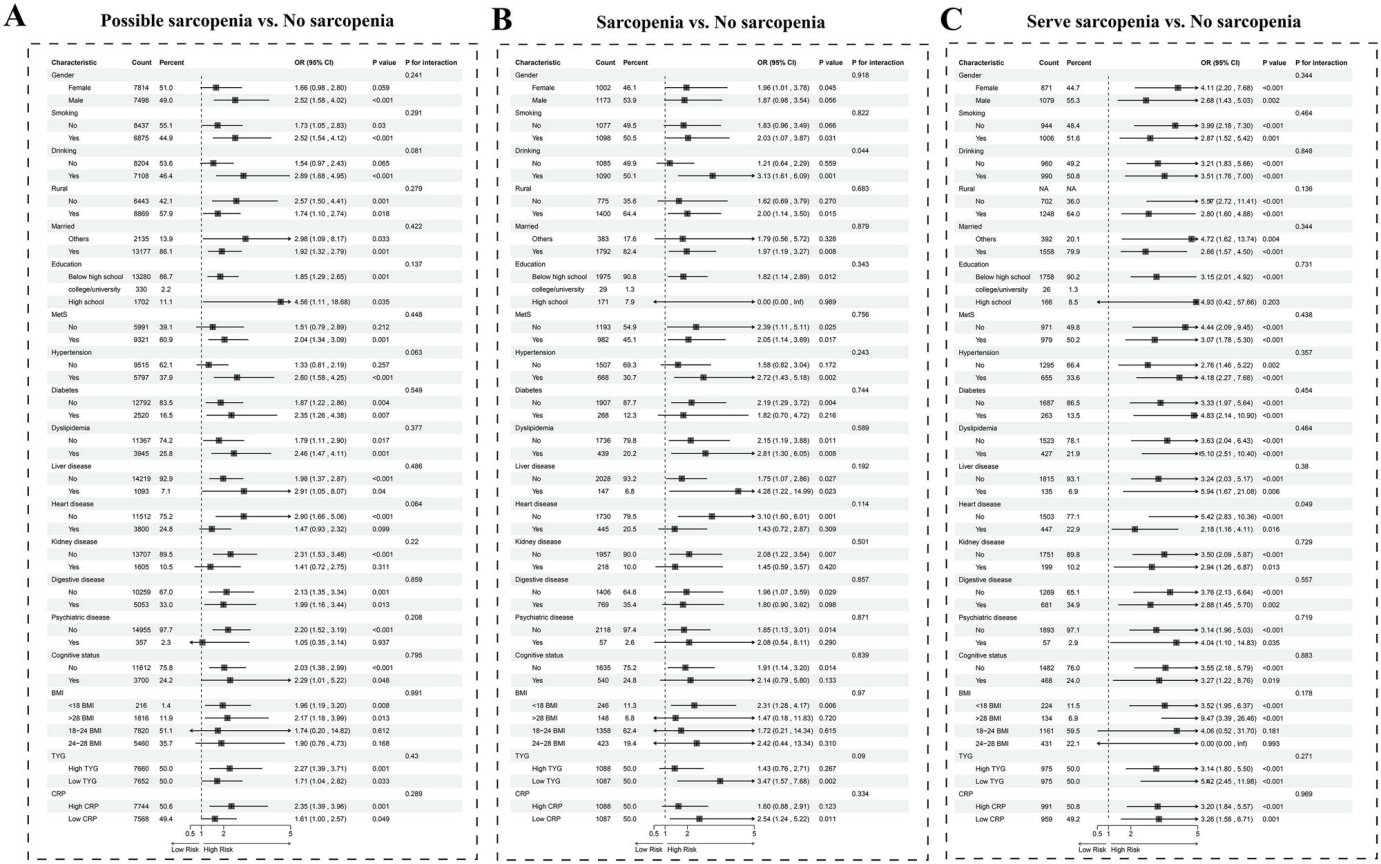
Subgroup analysis of the association between stroke and sarcopenia statuses. (A) Subgroup analysis estimating the association between stroke and possible sarcopenia. (B) Subgroup analysis estimating the association between stroke and sarcopenia. (C) Subgroup analysis estimating the association between stroke and severe sarcopenia. MetS, metabolic syndrome; BMI, body mass index; TyG, triglyceride‐glucose; CRP, C‐reactive protein; OR, odds ratio; CI, confidence interval.

Subgroup analyses were conducted in CHARLS across multiple factors, including gender, smoking, drinking, rural residence, marital status, education level, MetS, hypertension, diabetes, dyslipidemia, liver disease, heart disease, kidney disease, digestive disease, psychiatric disease, cognitive disease, BMI, triglyceride‐glucose (TyG) index, and CRP. These analyses confirmed that the increased risks of different sarcopenia statuses associated with stroke remained stable across most subgroups. Interaction analyses revealed minimal differences within subgroups, strengthening the robustness of our findings (Figure 3). Notably, the risk for severe sarcopenia was more obvious than for possible sarcopenia in subgroup analyses (Figure 3C).

The GEE model extended the generalized linear model (GLM) by accounting for the correction between observations, such as repeated measurements from the same subjects over time or data from individuals within the same group, and also, GEE worked by identifying a working correction structure to model the relationships between observations; with estimating equations, GEE focused on population‐averaged effects and helped to investigate longitudinal data. The model had advantages of flexibility, being suitable for many data types, robustness to misspecified correlation structures, and high computational efficiency (Lu et al. 2022). These sensitivity analyses based on the GEE model yielded consistent results demonstrating that stroke was significantly associated with increased risks of possible sarcopenia, sarcopenia, and severe sarcopenia in both CHARLS and HRS, except for sarcopenia in HRS, likely due to the limited number of confirmed cases (Table 4).

**TABLE 4:** Sensitivity analysis results with GEE model.

	CHARLS cohort results		
vs. no sarcopenia	Event/total individuals	Unadjusted model	
Possible sarcopenia	14,023/15,312	OR (95% CI)	*p*‐value
No stroke	13,278/14,533	1 (reference)	
Stroke	745/779	2.07 (1.46–2.93)	< 0.001
Sarcopenia	886/2175		
No stroke	842/2097	1 (reference)	
Stroke	44/78	1.93 (1.22–3.04)	0.005
Severe sarcopenia	661/1950		
No stroke	605/1860	1 (reference)	
Stroke	56/90	3.42 (2.21–5.29)	< 0.001
	**HRS cohort results**		
**vs. no sarcopenia**	**Event/Total individuals**	**Unadjusted model**	
Possible sarcopenia	2058/16,570	**OR (95% CI)**	***p*‐value**
No stroke	1637/15,041	1 (reference)	
Stroke	421/1529	3.11 (2.75–3.52)	< 0.001
Sarcopenia	18/14,530		
No stroke	15/13,419	1 (reference)	
Stroke	3/1111	2.42 (0.70–8.37)	0·163
Severe sarcopenia	104/14,616		
No stroke	88/13,492	1 (reference)	
Stroke	16/1124	2.20 (1.29–3.76)	0.004

Abbreviations: OR, odds ratio; 95% CI, 95% confidence interval.

## 4 Discussion

The present study elucidated significant demographic differences across sarcopenia statuses and between individuals with and without stroke. Among adults aged ≥ 50 years, stroke was associated with markedly elevated risks of possible sarcopenia, sarcopenia, and severe sarcopenia. In the CHARLS cohort, stroke patients exhibited increased risks of 107% for possible sarcopenia, 93% for sarcopenia, and 242% for severe sarcopenia. Similarly, in the HRS cohort, these risks were 211% for possible sarcopenia and 120% for severe sarcopenia. Mediation analyses identified self‐care ability (assessed via IADL score), nutritional status (BMI and eating disorders), and inflammation factors (CRP) as surrogate mediators in the stroke–sarcopenia relationship. Furthermore, RCS analyses quantified a nonlinear dose–response relationship between CRP levels and sarcopenia statuses. These findings characterized the stroke–sarcopenia association and highlighted underlying mechanisms, offering actionable insights for optimizing clinical management to avoid comorbidity like sarcopenia in stroke patients.

Previous research has primarily explored sarcopenia as a risk factor for CVD in which possible sarcopenia and sarcopenia were linked to 22% and 33% increased CVD risks, with even greater risks for stroke (Gao et al. 2022). Another prospective study underscored the high prevalence of sarcopenia among ischemic stroke patients, identifying it as an independent predictor of poor quality of life and prognosis (R. Chen et al. 2025). In our CHARLS cohort, stroke patients demonstrated a prevalence of 95.6% for possible sarcopenia, 56.4% for sarcopenia, and 62.2% for severe sarcopenia—substantially higher than rates reported by R. Chen et al. (2025). This disparity might stem from our study's focus on individuals aged ≥ 50 years and its large‐scale cohort design nature. Regarding the sarcopenia profile in poststroke patients, Huppertz et al. (2021) reported that malnutrition—closely associated with sarcopenia—increased over time to 19%, 52%, and 72% in the hyperacute, early subacute, and chronic phases of stroke, respectively. The overall prevalence of malnutrition was highest in rehabilitation settings (Cereda et al. 2016; Kaiser et al. 2010). Stroke patients experience rapid onset of muscle atrophy and muscle weakness immediately after the onset of the disease (Arasaki et al. 2009; Harris et al. 2001). Paralysis and associated disuse contribute to muscle weakness and skeletal muscle loss, which reduce physical activity and subsequently lead to the development or worsening of sarcopenia (Arasaki et al. 2009; Harris et al. 2001). A systematic review found that stroke patients spend more than 78% of their time in a sedentary state, regardless of time since stroke onset (Fini et al. 2017). Furthermore, impaired consciousness and dysphagia reduce nutritional intake, accelerating muscle loss and weakness. Patients with dysphagia are more likely to develop severe sarcopenia compared to those without (Maeda and Akagi 2015; Shimizu et al. 2021). The prevalence of sarcopenia in acute hospital settings after stroke has been reported to range from 8.5% to 33.8% (Sato et al. 2022; Bellelli et al. 2018), and sarcopenia is associated with unfavorable outcomes 90 days poststroke (Bellelli et al. 2018). In post‐acute rehabilitation hospitals, approximately 50% of stroke patients present with sarcopenia upon admission, which hinders functional recovery and reduces the likelihood of returning home (Shimizu et al. 2021, 2022; Nishioka et al. 2021). In a meta‐analysis, Inoue et al. (2022) reported that the prevalence of sarcopenia was 29.5% within the first 10 days after stroke and increased to 51.6% between 10 days and 1 month poststroke. This suggests that the risk of poststroke sarcopenia steadily increases over time (Inoue et al. 2022). Therefore, we recommend initiating risk screening and appropriate interventions for sarcopenia as early as possible after stroke.

Beyond establishing stroke as a risk factor, this study provides novel evidence of mediating pathways. In the HRS cohort, IADL accounted for 39.1% and 24.2% of the total effects on possible sarcopenia and severe sarcopenia, respectively, supporting prior findings that poststroke functional impairments such as difficulties with daily tasks exacerbated muscle loss and physical inactivity, thereby heightening sarcopenia risk (Batsis and Villareal 2018). This emphasized the critical role of rehabilitation programs aimed at restoring functional independence to mitigate sarcopenia risk in stroke survivors. Nutritional status also emerged as a key mediator: eating disorders, often linked to poststroke dysphagia or poor oral health, were associated with inadequate protein intake and accelerated muscle degradation (Treasure et al. 2020), while higher BMI mitigated sarcopenia and severe sarcopenia risks by −0.3% and −23.1%, respectively. This protective effect of BMI aligned with the concept of sarcopenia obesity, suggesting a nutritional reserve that counteracts muscle loss. Nutritional interventions such as dietary counseling, swallowing therapy, oral health care, and so forth might be necessary to prevent sarcopenia among stroke patients. Additionally, inflammation, proxied by CRP, mediated 7.1% and 16.8% of the total effects on possible sarcopenia and severe sarcopenia in CHARLS, respectively. RCS analyses revealed a nonlinear pattern, with elevated CRP levels beyond certain thresholds sharply increasing sarcopenia risk. In theory, poststroke pro‐inflammatory cytokines could activate catabolic pathways, promoting muscle protein breakdown and accelerating poststroke sarcopenia (Springer et al. 2014; Desgeorges et al. 2015; Scherbakov et al. 2013). Consequently, integrating anti‐inflammatory strategies such as targeted pharmacotherapy with nutritional and functional interventions could be beneficial to help reduce sarcopenia risk and improve long‐term outcomes in stroke survivors.

This study boasts several strengths. First, advanced machine learning techniques were employed to impute missing data, generating 10 matrices with 10 iterations each to preserve data integrity and maximize participant inclusion, thereby boosting the robustness and powered sample size of our findings. Second, this investigation was the first to examine the stroke–sarcopenia association across different sarcopenia statuses using both CHARLS and HRS cohorts, encompassing 33,551 participants, which was also the largest study of its kind to date. Moreover, mediation analyses and RCS modeling provided outstanding insights into mechanistic pathways and dose–response relationships, guiding potential interventions to prevent poststroke sarcopenia. Finally, subgroup and sensitivity analyses, including a reliable and flexible GEE framework, indicated the robustness and stability of our findings.

Nevertheless, limitations warrant consideration. As a cross‐sectional study, reverse causation between stroke and sarcopenia cannot be excluded. Longitudinal data are needed to establish temporality and track individual trajectories over time, inferring how exposures influence outcomes across multiple time points. Besides, cross‐sectional design could not capture better control for time‐variant confounders such as age and health‐related conditions (at different time points), and assessment of dynamic trends or long‐term effects, which were pivotal for chronic conditions like stroke and sarcopenia. The time‐variant exposure was not considered and analyzed, so residual confounding remained a concern. While robust evidence exhibits that the probability of sarcopenia events significantly expands in poststroke situation with time going on, therefore, we suggest adopting necessary measurements for sarcopenia risk screening as early as possible. Additionally, similar to other studies (Xie et al. 2019), stroke diagnoses relied on self‐reported physician‐diagnosed profiles due to the absence of medical records, limiting stroke subtypes identification and introducing potential recall bias; therefore, we analyzed all types of stroke together. However, some large‐scale populational studies such as based on English Longitudinal Study of Aging found that self‐reported onset of CVDs was strongly consistent with medical records (to 77.5% accuracy). Additionally, detailed information on stroke treatments was also lacking due to a self‐reported questionnaire. Because not all eligible participants had a strong medical background and knowledge, and the included participants with ≥ 50 years of age were likely to suffer memory loss, it was difficult for them to accurately report enough details on stroke treatment procedures as well as medications/drugs used, which inflamed selection and recall bias. Given that, we could not collect more detailed stroke treatment‐related data in both CHARLS and HRS. Despite rigorous imputation on missing data, discrepancies between the imputed and original data might have induced selection bias. Then, the current study still had an observational nature, which meant our results still provided observational relationship and might be biased by confounders. Lastly, while cross‐validation with the HRS cohort strengthened our findings, heterogeneity in measurements and diagnostic criteria between CHARLS and HRS may have influenced the result consistency.

## 5 Conclusion

This study demonstrated that stroke was associated with significantly increased risks of possible sarcopenia, sarcopenia, and severe sarcopenia among adults aged ≥ 50 years in both the CHARLS and HRS cohorts. Self‐care ability, nutritional status, and inflammation emerged as critical mediators, shedding light on the mechanisms linking stroke and sarcopenia. Our findings address the clinical imperative for comprehensive poststroke care, integrating rehabilitation to enhance functional independence, nutritional strategies to address eating disorders and optimize BMI, and anti‐inflammatory interventions to avert systemic inflammation. Such approaches hold promise for reducing sarcopenia risk and improving long‐term outcomes in stroke survivors. We recommend sarcopenia risk evaluation in poststroke populations using accessible measurements of muscle strength, muscle mass, and physical performance, along with the implementation of targeted interventions to address these modifiable mediators. Future prospective longitudinal studies are essential to confirm these associations and investigate stroke subtype‐specific impacts on sarcopenia risk, further refining clinical strategies.

## Author Contributions

**Xin Wang**: data curation, software, methodology, writing – original draft, writing – review and editing, formal analysis. **Jie Zhang**: data curation, writing – original draft, investigation, conceptualization, methodology, formal analysis, visualization. **Jing Xu**: conceptualization, investigation, writing – original draft, writing – review and editing, methodology, software, data curation, formal analysis, supervision, resources. **Ting Li**: conceptualization, methodology, software, data curation, supervision, writing—review and editing, writing—original draft, visualization, formal analysis, validation. **Qing Ye**: conceptualization, methodology, software, data curation, supervision, writing—review and editing, writing—original draft, visualization, validation, investigation, formal analysis, project administration, resources.

## Ethics Statements

All participants entered CHARLS and HRS cohorts have achieved ethical approval.

## Consent

Informed written consents were obtained from all participants.

## Conflicts of Interest

The authors declare no conflicts of interest.

## Peer Review

The peer review history for this article is available at https://publons.com/publon/10.1002/brb3.70763.

## Supporting information




**Supporting Table 1**: brb370763‐sup‐0001‐tableS1.docx

**Supporting Table 2**: brb370763‐sup‐0002‐tableS2.docx

**Supporting Table 3**: brb370763‐sup‐0003‐tableS3.docx

**Supporting Table 4**: brb370763‐sup‐0004‐tableS4.docx

## Data Availability

The datasets analyzed in the current study are public, and specific data are available from the corresponding author on reasonable request.
