# Supplementary material for: Insights Into the Association Between Stroke and Sarcopenia Risk in Adults Aged ≥ 50 Years: Cross‐Sectional Evidence From Two Large Population Longitudinal Cohorts
Source: Brain Behav. 2025 Aug 12;15(8):e70763. doi: 10.1002/brb3.70763 (PMC12340430; doi:10.1002/brb3.70763)
Supplement: Supplementary file 2 — Supporting Table 2: brb370763‐sup‐0002‐tableS2.docx [file BRB3-15-e70763-s001.docx]

**Supplementary Table 2.** Stroke-based patient characteristics for CHARLS

|  | **Overall (n = 16 859)** | **No stroke (n = 15 980)** | **Stroke (n = 879)** | **P-value** |  |
| --- | --- | --- | --- | --- | --- |
| Age (y) | 62.90± (9.17) | 62.70± (9.06) | 66.64± (10.22) | < 0.001 |  |
| Low muscle mass | 1 656 (9.8%) | 1 553 (9.7%) | 103 (12%) | 0.052 |  |
| Low muscle strength | 2 294 (14%) | 2 022 (13%) | 272 (31%) | < 0.001 |  |
| Low physical performance | 15 478 (92%) | 14 640 (92%) | 838 (95%) | < 0.001 |  |
| Chair stand test (s) | 10.02± (3.87) | 9.88± (3.75) | 12.50± (5.01) | < 0.001 |  |
| Frailty index | 18.44± (12.59) | 17.59± (11.86) | 33.84± (15.25) | < 0.001 |  |
| Left hand strength (kg) | 27.68± (9.84) | 27.92± (9.69) | 23.43± (11.46) | < 0.001 |  |
| Right hand strength (kg) | 29.06± (10.23) | 29.25± (10.16) | 25.59± (10.95) | < 0.001 |  |
| BMI (kg/m2) | 24.59± (23.14) | 24.57± (23.59) | 25.06± (12.44) | < 0.001 |  |
| Total MET (MET-hour/week) | 6 340.00± (5 153.70) | 6 419.30± (5 158.09) | 4 898.35± (4 855.24) | < 0.001 |  |
| Height (m) | 1.58± (0.09) | 1.58± (0.09) | 1.57± (0.10) | 0.600 |  |
| Waist (cm) | 85.48± (11.97) | 85.37± (11.95) | 87.47± (12.33) | < 0.001 |  |
| Weight (kg) | 59.41± (10.97) | 59.35± (10.94) | 60.52± (11.36) | 0.001 |  |
| Systolic pressure (mmHg) | 128.54± (18.72) | 128.16± (18.59) | 135.57± (19.66) | < 0.001 |  |
| TYG | 8.72± (0.54) | 8.71± (0.54) | 8.85± (0.58) | < 0.001 |  |
| TYG BMI | 213.34± (120.91) | 212.71± (121.26) | 224.84± (113.72) | < 0.001 |  |
| Speed | 1.31± (6.26) | 1.33± (6.42) | 0.91± (1.46) | < 0.001 |  |
| Total cholesterol (mg/dl) | 185.04± (31.01) | 185.03± (31.01) | 185.30± (30.91) | 0.510 |  |
| TG (mg/dl) | 140.46± (74.41) | 139.90± (74.14) | 150.65± (78.56) | < 0.001 |  |
| HDL cholesterol (mg/dl) | 51.44± (9.89) | 51.56± (9.89) | 49.22± (9.72) | < 0.001 |  |
| LDL cholesterol (mg/dl) | 103.45± (24.61) | 103.40± (24.61) | 104.31± (24.55) | 0.150 |  |
| CRP | 2.86± (5.20) | 2.80± (5.08) | 3.96± (7.06) | < 0.001 |  |
| HbA1c (%) | 6.01± (0.87) | 6.00± (0.86) | 6.31± (1.05) | < 0.001 |  |
| Gender |  |  |  | 0.001 |  |
| Female | 8 597 (51%) | 8 198 (51%) | 399 (45%) |  |  |
| Male | 8 262 (49%) | 7 782 (49%) | 480 (55%) |  |  |
| Hypertension | 6 260 (37%) | 5 668 (35%) | 592 (67%) | < 0.001 |  |
| Dyslipidemia | 4 143 (25%) | 3 697 (23%) | 446 (51%) | < 0.001 |  |
| Diabetes | 2 673 (16%) | 2 341 (15%) | 332 (38%) | < 0.001 |  |
| Liver disease**^a^** | 1 183 (7.0%) | 1 051 (6.6%) | 132 (15%) | < 0.001 |  |
| Heart disease | 4 104 (24%) | 3 690 (23%) | 414 (47%) | < 0.001 |  |
| Kidney disease**^a^** | 1 778 (11%) | 1 575 (9.9%) | 203 (23%) | < 0.001 |  |
| Digestive disease | 5 655 (34%) | 5 303 (33%) | 352 (40%) | < 0.001 |  |
| Psychiatric disease | 413 (2.4%) | 344 (2.2%) | 69 (7.8%) | < 0.001 |  |
| Cognitive status | 4 156 (25%) | 3 959 (25%) | 197 (22%) | 0.110 |  |
| Headache | 2 514 (15%) | 2 283 (14%) | 231 (26%) | < 0.001 |  |
| Eating disorders | 521 (3.1%) | 385 (2.4%) | 136 (15%) | < 0.001 |  |
| Rural living | 10 043 (60%) | 9 575 (60%) | 468 (53%) | < 0.001 |  |
| Married |  |  |  | < 0.001 |  |
| Others | 2 612 (15%) | 2 418 (15%) | 194 (22%) |  |  |
| Yes | 14 247 (85%) | 13 562 (85%) | 685 (78%) |  |  |
| Drinking | 7 822 (46%) | 7 396 (46%) | 426 (48%) | 0.210 |  |
| Smoking | 7 651 (45%) | 7 203 (45%) | 448 (51%) | 0.001 |  |
| Education |  |  |  | 0.240 |  |
| Below high school | 14 787 (88%) | 14 025 (88%) | 762 (87%) |  |  |
| College/university | 333 (2.0%) | 309 (1.9%) | 24 (2.7%) |  |  |
| High school | 1 739 (10%) | 1 646 (10%) | 93 (11%) |  |  |
| MetS | 9 960 (59%) | 9 263 (58%) | 697 (79%) | < 0.001 |  |
| Diabetes medication use | 2 445 (15%) | 2 121 (13%) | 324 (37%) | < 0.001 |  |
| Digestive medication use | 4 149 (25%) | 3 880 (24%) | 269 (31%) | < 0.001 |  |
| Dyslipidemia medication use | 2 875 (17%) | 2 503 (16%) | 372 (42%) | < 0.001 |  |
| Heart disease medication use | 3 439 (20%) | 3 081 (19%) | 358 (41%) | < 0.001 |  |
| Hypertension medication use | 5 268 (31%) | 4 729 (30%) | 539 (61%) | < 0.001 |  |
| Stroke medication use | 766 (4.5%) | 40 (0.3%) | 726 (83%) | < 0.001 |  |
| **^a^**Indicate chronic diseases.  **Abbreviations**: BMI, body mass index; MET, metabolic equivalent of task; TYG, triglyceride-glucose index; TG, triglyceride; HDL, high-density lipoprotein cholesterol; LDL, low-density lipoprotein cholesterol; CRP, C-reactive protein; MetS, metabolism syndrome. | | | | |  |
|  |  |  |  |  |  |
