# Supplementary material for: Insights Into the Association Between Stroke and Sarcopenia Risk in Adults Aged ≥ 50 Years: Cross‐Sectional Evidence From Two Large Population Longitudinal Cohorts
Source: Brain Behav. 2025 Aug 12;15(8):e70763. doi: 10.1002/brb3.70763 (PMC12340430; doi:10.1002/brb3.70763)
Supplement: Supplementary file 3 — Supporting Table 3: brb370763‐sup‐0003‐tableS3.docx [file BRB3-15-e70763-s003.docx]

**Supplementary Table 3.** Stroke-based patient characteristics for HRS

|  | **Overall (N = 16 692)** | **No stroke (N = 15 144)** | **Stroke (N = 1 548)** | **P-value** |  |
| --- | --- | --- | --- | --- | --- |
| Age | 67.60± (10.89) | 67.06± (10.69) | 72.90± (11.41) | < 0.001 |  |
| Low muscle mass | 331 (2.0%) | 299 (2.0%) | 32 (2.1%) | 0.800 |  |
| Low muscle strength | 2 180 (13%) | 1 740 (11%) | 440 (28%) | < 0.001 |  |
| Low physical performance | 12 246 (73%) | 10 973 (72%) | 1 273 (82%) | < 0.001 |  |
| Education year | 12.86± (3.25) | 12.91± (3.27) | 12.42± (3.05) | < 0.001 |  |
| ADL | 0.47± (1.18) | 0.39± (1.06) | 1.25± (1.84) | < 0.001 |  |
| Cigarette (number/day) | 1.44± (4.67) | 1.42± (4.65) | 1.54± (4.87) | 0.120 |  |
| Exercise frequency (times/week) | 4.43± (1.66) | 4.35± (1.64) | 5.22± (1.58) | < 0.001 |  |
| Left hand strength (kg) | 27.24± (10.55) | 27.55± (10.42) | 24.24± (11.27) | < 0.001 |  |
| Right hand strength (kg) | 32.69± (9.98) | 32.89± (10.01) | 30.65± (9.46) | < 0.001 |  |
| BMI (kg/m2) | 30.50± (4.09) | 30.46± (4.12) | 30.92± (3.82) | < 0.001 |  |
| Height (m) | 1.65± (0.10) | 1.66± (0.10) | 1.65± (0.10) | < 0.001 |  |
| Weight (kg) | 83.78± (14.46) | 83.75± (14.50) | 84.02± (14.04) | 0.330 |  |
| Waist (cm) | 41.47± (5.45) | 41.34± (5.49) | 42.75± (4.87) | < 0.001 |  |
| IADL | 0.35± (0.95) | 0.29± (0.83) | 1.00± (1.56) | < 0.001 |  |
| Systolic pressure (mmHg) | 127.33± (13.53) | 127.17± (13.55) | 128.90± (13.17) | < 0.001 |  |
| Diastolic pressure (mmHg) | 77.19± (8.26) | 77.32± (8.24) | 75.92± (8.33) | < 0.001 |  |
| speed (m/s) | 0.77± (0.51) | 0.78± (0.51) | 0.69± (0.46) | < 0.001 |  |
| Married |  |  |  | < 0.001 |  |
| Others | 7 619 (46%) | 6 762 (45%) | 857 (55%) |  |  |
| Yes | 9 073 (54%) | 8 382 (55%) | 691 (45%) |  |  |
| Gender |  |  |  | 0.200 |  |
| Female | 9 765 (59%) | 8 883 (59%) | 882 (57%) |  |  |
| Male | 6 927 (41%) | 6 261 (41%) | 666 (43%) |  |  |
| Rural living |  |  |  | < 0.001 |  |
| No | 9 189 (55%) | 8 415 (56%) | 774 (50%) |  |  |
| Others | 3 598 (22%) | 3 211 (21%) | 387 (25%) |  |  |
| Yes | 3 905 (23%) | 3 518 (23%) | 387 (25%) |  |  |
| Smoking | 8 996 (54%) | 8 037 (53%) | 959 (62%) | < 0.001 |  |
| Drinking | 9 358 (56%) | 8 737 (58%) | 621 (40%) | < 0.001 |  |
| Hypertension | 10 540 (63%) | 9 246 (61%) | 1 294 (84%) | < 0.001 |  |
| Diabetes | 4 822 (29%) | 4 189 (28%) | 633 (41%) | < 0.001 |  |
| Cancer | 2 578 (15%) | 2 260 (15%) | 318 (21%) | < 0.001 |  |
| Heart disease | 4 173 (25%) | 3 366 (22%) | 807 (52%) | < 0.001 |  |
| Psychiatric disease | 3 030 (18%) | 2 596 (17%) | 434 (28%) | < 0.001 |  |
| Headache | 1 396 (8.4%) | 1 015 (6.7%) | 381 (25%) | < 0.001 |  |
| Fatigue | 3 046 (18%) | 2 043 (13%) | 1 003 (65%) | < 0.001 |  |
| Angina | 1 486 (8.9%) | 1 131 (7.5%) | 355 (23%) | < 0.001 |  |
| Congestive heart failure | 1 258 (7.5%) | 933 (6.2%) | 325 (21%) | < 0.001 |  |
| Arrhythmia | 2 824 (17%) | 2 251 (15%) | 573 (37%) | < 0.001 |  |
| Dyslipidemia | 9 862 (59%) | 8 732 (58%) | 1 130 (73%) | < 0.001 |  |
| Hypertension medication use | 9 202 (55%) | 8 007 (53%) | 1 195 (77%) | < 0.001 |  |
| Diabetes medication use | 3 468 (21%) | 2 987 (20%) | 481 (31%) | < 0.001 |  |
| Stroke medication use | 493 (3.0%) | 4 (<0.1%) | 489 (32%) | < 0.001 |  |
| Angina medication use | 991 (5.9%) | 740 (4.9%) | 251 (16%) | < 0.001 |  |
| Congestive heart failure medication use | 832 (5.0%) | 620 (4.1%) | 212 (14%) | < 0.001 |  |
| Psychiatric medication use | 1 787 (11%) | 1 532 (10%) | 255 (16%) | < 0.001 |  |
| Heart disease medication use | 306 (1.8%) | 218 (1.4%) | 88 (5.7%) | < 0.001 |  |
| Dyslipidemia medication use | 7 231 (43%) | 6 259 (41%) | 972 (63%) | < 0.001 |  |
| Digestive medication use | 2 893 (17%) | 2 480 (16%) | 413 (27%) | < 0.001 |  |
| Physical activity use | 6 506 (39%) | 6 047 (40%) | 459 (30%) | < 0.001 |  |
| Obesity | 7 445 (45%) | 6 689 (44%) | 756 (49%) | < 0.001 |  |
| **Abbreviations**: ADL, activities of daily living; BMI, body mass index; IADL, instrumental activities of daily living. | | | | |  |
|  |  |  |  |  |  |
