# Supplementary material for: Insights Into the Association Between Stroke and Sarcopenia Risk in Adults Aged ≥ 50 Years: Cross‐Sectional Evidence From Two Large Population Longitudinal Cohorts
Source: Brain Behav. 2025 Aug 12;15(8):e70763. doi: 10.1002/brb3.70763 (PMC12340430; doi:10.1002/brb3.70763)
Supplement: Supplementary file 4 — Supporting Table 4: brb370763‐sup‐0004‐tableS4.docx [file BRB3-15-e70763-s002.docx]

**Supplementary Table 4.** Stepwise regression analysis results

| **Possible sarcopenia vs. No sarcopenia (covariables included in model 4)** | | | | |  | **Possible sarcopenia vs. No sarcopenia (covariables included in model 4)** | | | | |  |
| --- | --- | --- | --- | --- | --- | --- | --- | --- | --- | --- | --- |
| **Variable** | **Estimate** | **Std. Error** | **z value** | **Pr(>\|z\|)** |  | **Variable** | **Estimate** | **Std. Error** | **z value** | **Pr(>\|z\|)** |  |
| (Intercept) | 4.21894122 | 0.262493358 | 16.07256372 | 3.97309E-58 |  | (Intercept) | -9.319425721 | 0.357462414 | -26.07106468 | 7.7638E-150 |  |
| Stroke (yes vs. no) | 0.627246961 | 0.181623536 | 3.453555497 | 0.000553248 |  | Stroke (yes vs. no) | 0.203671743 | 0.081855559 | 2.488184613 | 0.012839706 |  |
| Age | -0.027703022 | 0.003581077 | -7.735947293 | 1.02636E-14 |  | Age | 0.102760918 | 0.002864974 | 35.86800837 | 9.6366E-282 |  |
| Gender (male vs. female) | -0.221622769 | 0.069794864 | -3.175344943 | 0.001496585 |  | Gender (male vs. female) | 0.275352471 | 0.057217658 | 4.812368743 | 1.49152E-06 |  |
| Marital status (yes vs. no) | -0.297107297 | 0.096331503 | -3.084217391 | 0.002040884 |  | BMI | -0.025464258 | 0.007705569 | -3.304656439 | 0.000950929 |  |
| Drinking (yes vs. no) | -0.139758889 | 0.06767877 | -2.065032927 | 0.038919885 |  | Education year | -0.025496916 | 0.00879719 | -2.898302436 | 0.003751886 |  |
| MetS (yes vs. no) | 0.338286886 | 0.069141776 | 4.892655456 | 9.94845E-07 |  | Smoking (yes vs. no) | -0.116736078 | 0.0565672 | -2.063670769 | 0.039048943 |  |
| Eating difficulties (yes vs. no) | 1.320538286 | 0.341396465 | 3.868049088 | 0.00010971 |  | Drinking (yes vs. no) | -0.1510271 | 0.058013245 | -2.603321005 | 0.009232546 |  |
| Dyslipidemia (yes vs. no) | -0.22909433 | 0.071468331 | -3.205536289 | 0.00134811 |  | Psychiatric disease (yes vs. no) | 0.129156366 | 0.076155636 | 1.695952831 | 0.089894813 |  |
| CRP | 0.04804098 | 0.012250896 | 3.921425946 | 8.80265E-05 |  | Fatigue (yes vs. no) | 0.349369004 | 0.071288718 | 4.900761514 | 9.54659E-07 |  |
| Hypertension medication use (yes vs. no) | 0.170408442 | 0.077207755 | 2.207141507 | 0.027304175 |  | Arrhythmia (yes vs. no) | 0.097631014 | 0.063626934 | 1.534429033 | 0.12492414 |  |
|  |  |  |  |  |  | IADL | 0.289059024 | 0.023638883 | 12.22811698 | 2.19979E-34 |  |
| **Sarcopenia vs. No sarcopenia (covariables included in model 4)** | | | | |  | Exercise frequency | 0.146159896 | 0.019848264 | 7.363863096 | 1.78663E-13 |  |
| **Variable** | **Estimate** | **Std. Error** | **z value** | **Pr(>\|z\|)** |  | Diabetes (yes vs. no) | 0.13648222 | 0.059057403 | 2.311009515 | 0.020832329 |  |
| (Intercept) | -6.749479425 | 0.725060756 | -9.308846697 | 1.29228E-20 |  |  |  |  |  |  |  |
| Stroke (yes vs. no) | 0.657595588 | 0.281148818 | 2.338959108 | 0.01933755 |  | **Sarcopenia vs. No sarcopenia (covariables included in model 4)** | | | | |  |
| Age | 0.120883048 | 0.007875659 | 15.34894374 | 3.6E-53 |  | **Variable** | **Estimate** | **Std. Error** | **z value** | **Pr(>\|z\|)** |  |
| Gender (male vs. female) | -0.302624104 | 0.121849783 | -2.483583449 | 0.013006786 |  | (Intercept) | -3.567623522 | 2.72563794 | -1.308913216 | 0.190563749 |  |
| Marital status (yes vs. no) | -0.444891038 | 0.143586884 | -3.098410012 | 0.00194562 |  | Stroke (yes vs. no) | 1.346391091 | 0.710546099 | 1.894868036 | 0.058109887 |  |
| Rural residence (yes vs. no) | 0.597356494 | 0.113387747 | 5.268263206 | 1.37721E-07 |  | Age | 0.111267074 | 0.029671363 | 3.749981829 | 0.000176847 |  |
| Education (college/university vs. below high school) | -1.432022363 | 0.669953047 | -2.137496606 | 0.032557621 |  | BMI | -0.595786714 | 0.073221142 | -8.136812624 | 4.0582E-16 |  |
| Education (high school vs. below high school) | -1.038587816 | 0.257643009 | -4.03111196 | 5.55136E-05 |  | Marital status (yes vs. no) | 0.806998604 | 0.560177088 | 1.440613372 | 0.14969394 |  |
| Drinking (yes vs. no) | -0.182713364 | 0.116177051 | -1.572714768 | 0.115784879 |  | Education year | 0.251694715 | 0.109922368 | 2.289749757 | 0.022035827 |  |
| MetS (yes vs. no) | -0.821510348 | 0.1244272 | -6.602337311 | 4.04725E-11 |  | Drinking (yes vs. no) | -1.031642821 | 0.551290754 | -1.871322555 | 0.061300386 |  |
| Eating difficulties (yes vs. no) | 1.041680322 | 0.440003541 | 2.367436222 | 0.017911807 |  | Cancer (yes vs. no) | 1.260629617 | 0.533001623 | 2.365151556 | 0.018022694 |  |
| Dyslipidemia (yes vs. no) | -0.764014025 | 0.226328519 | -3.375686047 | 0.000736319 |  |  |  |  |  |  |  |
| Psychiatric disease (yes vs. no) | 0.495908053 | 0.317824034 | 1.560322695 | 0.118683643 |  | **Severe sarcopenia vs. No sarcopenia (covariables included in model 4)** | | | | |  |
| Cognitive status (yes vs. no) | 0.553285743 | 0.120750493 | 4.58205782 | 4.60423E-06 |  | **Variable** | **Estimate** | **Std. Error** | **z value** | **Pr(>\|z\|)** |  |
| CRP | 0.043683104 | 0.013380746 | 3.264623846 | 0.001096095 |  | (Intercept) | -2.856420439 | 1.197331083 | -2.385656297 | 0.017048676 |  |
| HbA1c | -0.20654066 | 0.082487746 | -2.50389507 | 0.012283446 |  | Age | 0.158099051 | 0.014811926 | 10.67376719 | 1.35038E-26 |  |
| Hypertension (yes vs. no) | -0.424169049 | 0.183232574 | -2.31492163 | 0.020617221 |  | Gender (male vs. female) | -0.749025175 | 0.343485796 | -2.180658367 | 0.029208695 |  |
| Digestive medication use (yes vs. no) | 0.377069221 | 0.117686589 | 3.204011809 | 0.001355269 |  | BMI | -0.583019861 | 0.036609406 | -15.92541157 | 4.22226E-57 |  |
| Dyslipidemia medication use (yes vs. no) | 0.443642943 | 0.27018066 | 1.642023315 | 0.100585172 |  | Fatigue (yes vs. no) | 0.473024573 | 0.314097675 | 1.505979222 | 0.132072508 |  |
| Hypertension medication use (yes vs. no) | 0.311079117 | 0.206855162 | 1.50384991 | 0.132620017 |  | IADL | 0.2676827 | 0.134835003 | 1.985261197 | 0.047115419 |  |
|  |  |  |  |  |  | Exercise | 0.207555705 | 0.067108516 | 3.09283705 | 0.00198253 |  |
| **Severe sarcopenia vs. No sarcopenia (covariables included in model 4)** | | | | |  | Diabetes (yes vs. no) | 0.470053147 | 0.318649659 | 1.475140907 | 0.140174665 |  |
| **Variable** | **Estimate** | **Std. Error** | **z value** | **Pr(>\|z\|)** |  |  |  |  |  |  |  |
| (Intercept) | -14.05328747 | 0.843459916 | -16.66147639 | 2.49782E-62 |  |  |  |  |  |  |  |
| Stroke (yes vs. no) | 0.844219436 | 0.337874207 | 2.498620544 | 0.012467773 |  |  |  |  |  |  |  |
| Age | 0.204844241 | 0.010742778 | 19.06808771 | 4.65001E-81 |  |  |  |  |  |  |  |
| Marital status (yes vs. no) | -0.561507969 | 0.172653393 | -3.252226664 | 0.001145047 |  |  |  |  |  |  |  |
| Rural residence (yes vs. no) | 0.799002112 | 0.15292682 | 5.22473501 | 1.74405E-07 |  |  |  |  |  |  |  |
| Education (college/university vs. below high school) | -14.85736692 | 409.5465417 | -0.036277603 | 0.971061008 |  |  |  |  |  |  |  |
| Education (high school vs. below high school) | -0.649614084 | 0.321845908 | -2.018400945 | 0.043549521 |  |  |  |  |  |  |  |
| Drinking (yes vs. no) | -0.298486742 | 0.1369945 | -2.178822823 | 0.029344833 |  |  |  |  |  |  |  |
| MetS (yes vs. no) | -0.342543959 | 0.150703083 | -2.272972472 | 0.023027839 |  |  |  |  |  |  |  |
| Eating difficulties (yes vs. no) | 2.158780355 | 0.462291697 | 4.669736381 | 3.01586E-06 |  |  |  |  |  |  |  |
| Dyslipidemia (yes vs. no) | -0.51557012 | 0.186640735 | -2.762366535 | 0.005738402 |  |  |  |  |  |  |  |
| Kidney disease (yes vs. no) | 0.368359389 | 0.228200027 | 1.614195207 | 0.106485091 |  |  |  |  |  |  |  |
| Cognitive status (yes vs. no) | 1.032631458 | 0.158802768 | 6.502603643 | 7.89416E-11 |  |  |  |  |  |  |  |
| Systolic | -0.007897774 | 0.003694464 | -2.137731866 | 0.032538512 |  |  |  |  |  |  |  |
| CRP | 0.071636282 | 0.015761538 | 4.545005756 | 5.49338E-06 |  |  |  |  |  |  |  |
| Digestive medication use (yes vs. no) | 0.539239395 | 0.153390447 | 3.515469219 | 0.000438978 |  |  |  |  |  |  |  |
|  |  |  |  |  |  |  |  |  |  |  |  |
|  | | | | | | | | | | |  |
|  |  |  |  |  |  |  |  |  |  |  |  |
